# 614. Epidemiology of three vaccine-preventable infectious diseases within United States immigration detention centers, 2019 through 2023

**DOI:** 10.1093/ofid/ofaf695.187

**Published:** 2026-01-11

**Authors:** Ribhav Gupta, Dean L Winslow, Ronit Gupta, Sten Vermund

**Affiliations:** Stanford University, Stanford, CA; Stanford University, Stanford, CA; Harvard University, Boston, Massachusetts; University of South Florida, Tampa, Florida

## Abstract

**Background:**

Migrants detained by U.S. Immigration and Customs Enforcement (ICE) are thought to have a high risk of preventable infectious diseases due to crowding and poor healthcare access. Prior studies had limited data access and none report post-pandemic trends. We assessed epidemiologic patterns of three vaccine-preventable diseases across ICE facilities.

**Figure:**
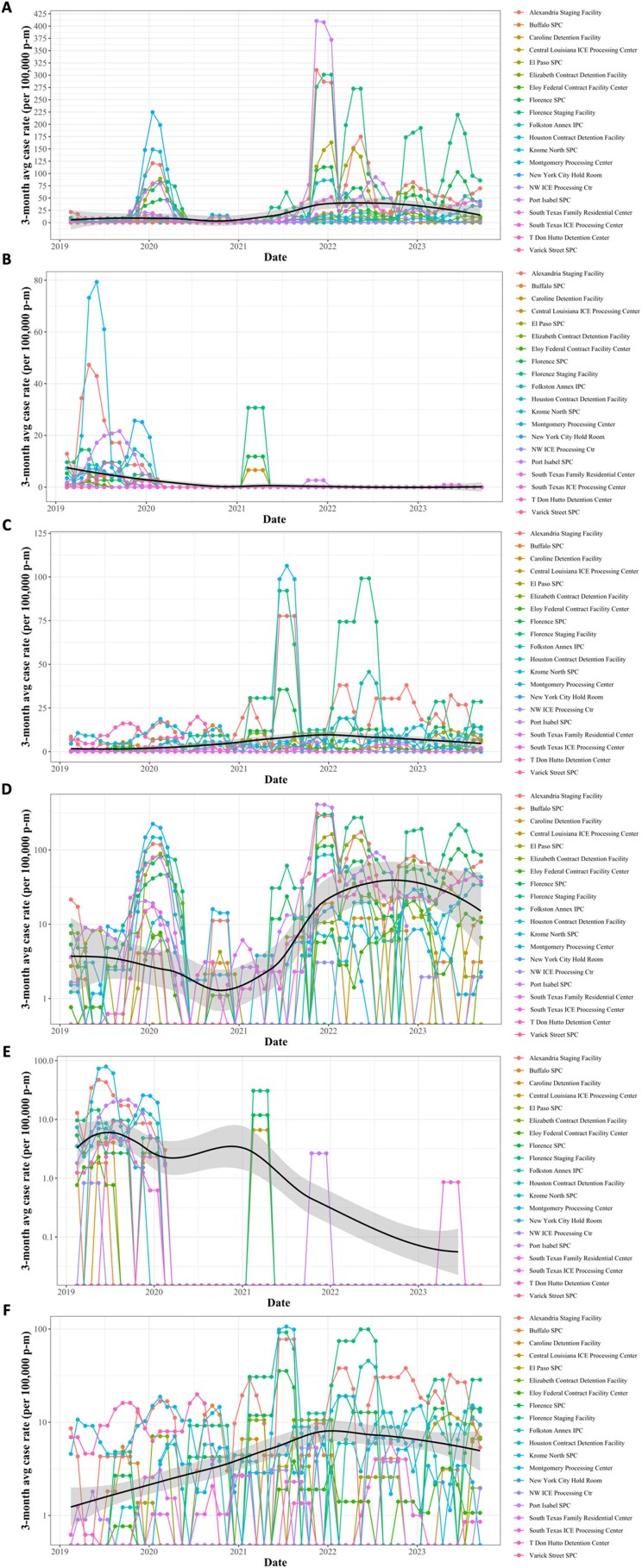
Three month sliding average of case rate (per 100,000 person-months) over time stratified by reporting detention facility from 2019 through 2023.

**Figure:**
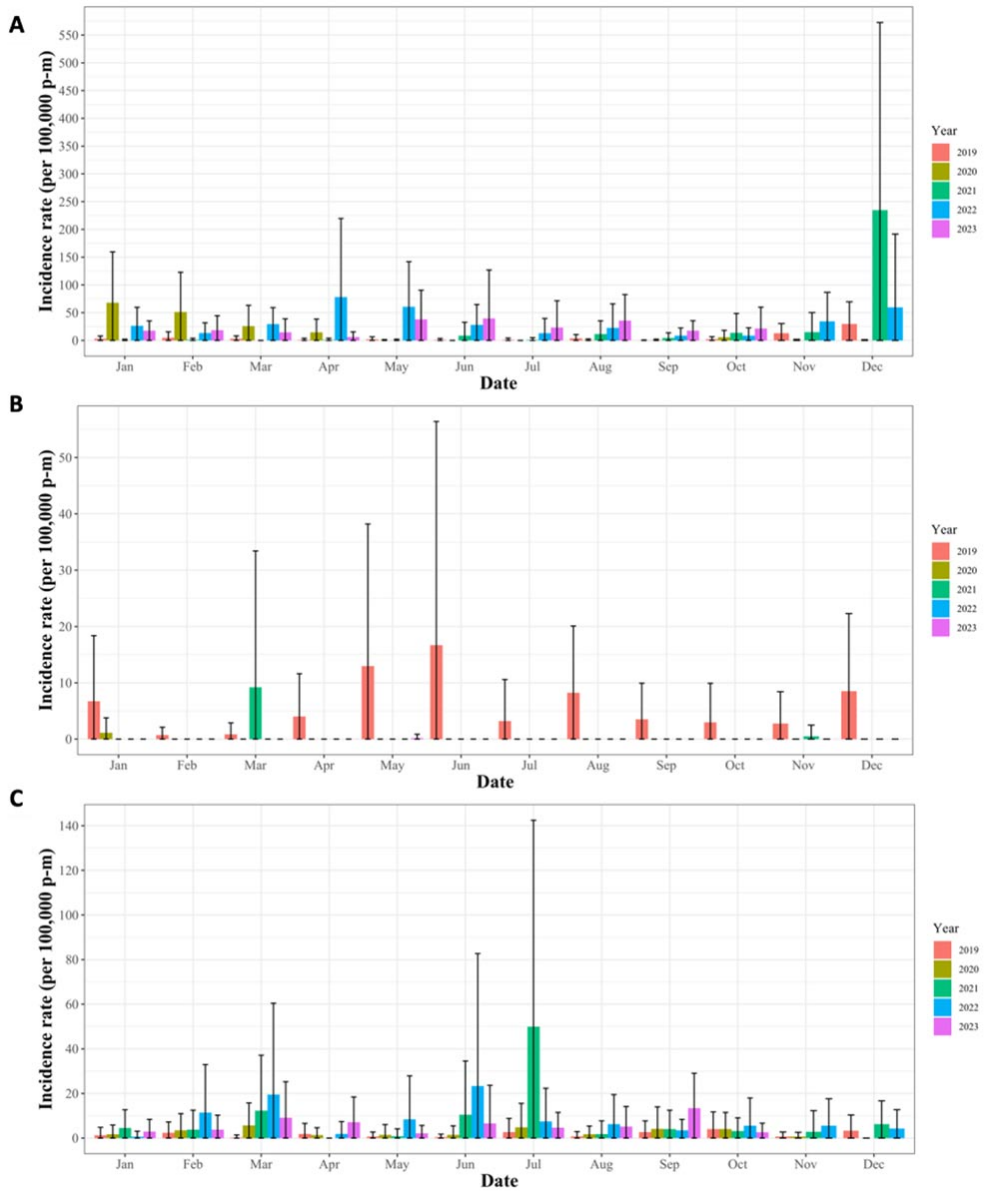
Seasonal analysis of variations in facility-level case rate by month from 2019 through 2023. Bar plots of mean facility-level incidence per year and grouped by month. Panel A. Case rate of influenza; Panel B. Case rate of mumps; Panel C. Case rate of hepatitis A. Note: Variation in Y-axis scale dependent on disease panel. Panel A. Case rate of influenza on linear scale; Panel B. Case rate of mumps on linear scale; Panel C. Case rate of hepatitis A on linear scale; Panel D. Case rate of influenza on log scale; Panel E. Case rate of mumps on log scale; Panel F. Case rate of hepatitis A on log scale. Black line is mean facility-level case rate with a shadow of the standard deviation. Note: Variation in Y-axis scale dependent on disease panel. Y-axis axis on log scale.

**Methods:**

We obtained ICE detainee data on influenza, mumps, and hepatitis A from January 2019–October 2023 from the Department of Homeland Security for 20 centers. National influenza data came from CDC FluView. Case counts were aggregated monthly, and case rates estimated using annualized average daily populations. Outbreaks were defined as clusters of three or more monthly cases within a facility. We characterized case demographics, system- and facility-level trends, seasonality, outbreaks, and geospatial patterns.

**Figure:**
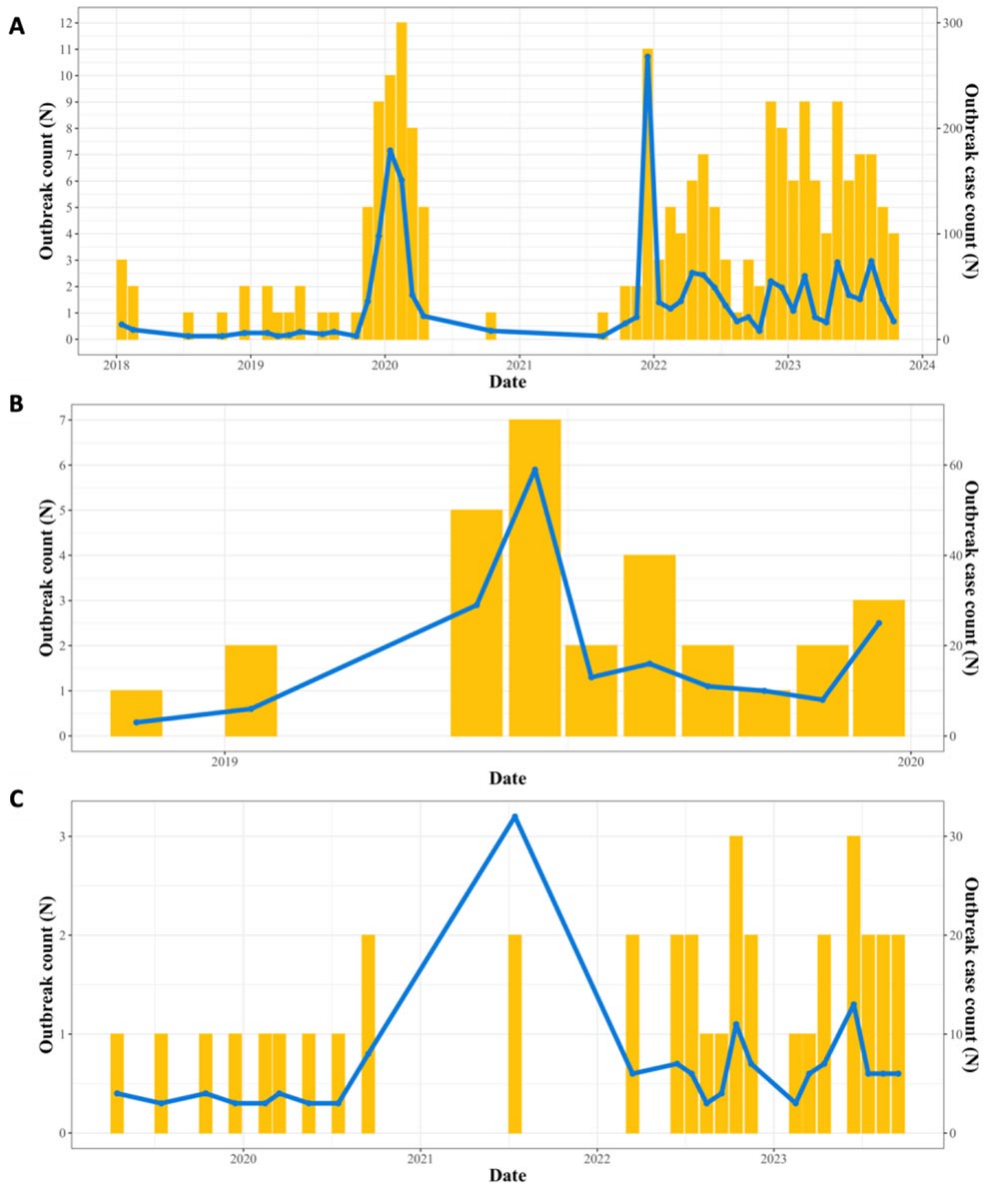
Number of outbreaks and outbreak cases over time across the system from 2019 through 2023.

**Figure:**
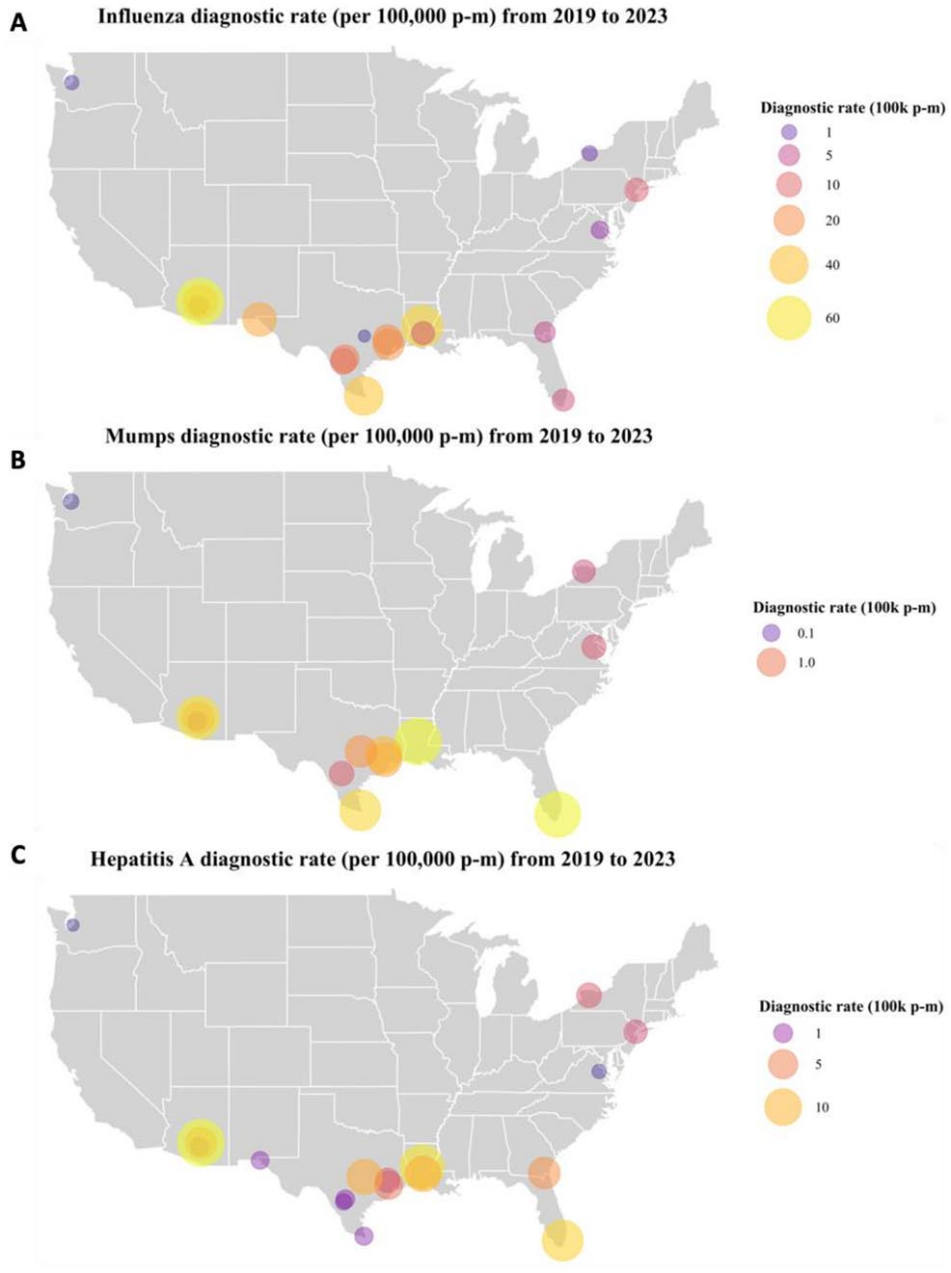
Bar plot of number of facilities with active outbreaks per month and line plot of number of system-level outbreak cases over time. Panel A. Case rate of influenza; Panel B. Case rate of mumps; Panel C. Case rate of hepatitis A. Note: Variation in X-axis and Y-axis scale dependent on disease panel. Spatial distribution of average case rate (per 100,000 person-months) from 2019 through 2023 at facility level. Choropleth map of averaged case rate per disease at facility level with circle size and color corresponding to case rate. Panel A. Case rate of influenza; Panel B. Case rate of mumps; Panel C. Case rate of hepatitis A. Note: Variation in legend.

**Results:**

From 2019–2023, 2,035 influenza cases were reported, averaging 35.1 cases monthly (range: 0–276). The average facility-level case rate was 19.4 cases per 100,000 person-months [P-M]; range: 0–1009.1). December had the highest average facility-level case rate (25.6 cases) and July the lowest (2.7), paralleling national trends. Across 15 facilities, 79 outbreaks occurred involving 1,739 cases with an average duration of 2.5 months (range: 1–13). For mumps, 252 reported cases averaged 4.3 monthly (range: 0–61). The average facility-level case rate (per 100,000 p-m) was 1.4 (range: 0–160.2). June had the highest average facility-level rate (1.3) and February the lowest (0.8); 16 outbreaks across 8 facilities involved 177 with an average duration of 1.8 months (range: 1–6). For hepatitis A, 486 cases were reported, averaging 8.4 monthly (range: 0–40). The average facility-level rate (per 100,000 p-m) was 5.2 (range: 0–273.4). July had the highest average facility-level case rate (5.5) and November the lowest (0.8); 33 outbreaks across 11 facilities involved 158 cases, averaging 1.2 months (range: 1–2). No geospatial clusters were observed.

**Conclusion:**

ICE detainees have high rates of vaccine-preventable infectious diseases with wide variation across facilities. ICE vaccination campaigns and improved facility procedures could reduce disease burden, protecting migrant and staff health.

**Disclosures:**

All Authors: No reported disclosures

